# Supplementary material for: Factors affecting the biology of Pachycrepoideus vindemmiae (Hymenoptera: Pteromalidae), a parasitoid of spotted-wing drosophila (Drosophila suzukii)
Source: PLoS One. 2019 Jul 23;14(7):e0218301. doi: 10.1371/journal.pone.0218301 (PMC6650059; doi:10.1371/journal.pone.0218301)
Supplement: S1 Table — Pupae of spotted-wing drosophila (Drosophila suzukii) (Diptera: Drosophilidae) were used as hosts. The numbers of replicates respectively for water, honey, water + honey, and fasting in the assay #1 were 10, 8, 9, and 8 for males; 13, 14, 16, and 10 for females; and 11, 14, 10, and 12 for females + hosts. In the assay #2 the number of replicates were 94 (water), 47 (honey), 95 (water + honey), 39 (no water, no honey), and 96 (host, SWD pupa). In both assays each replicate was formed by one mated individual wasp. (DOCX) [file pone.0218301.s001.docx]

**S1 Table**.

| **Group** | **Contrast^1^** | **χ^2^** | **DF** | **P-value** |
| --- | --- | --- | --- | --- |
| **Assay #1 – Constant supply** |  |  |  |  |
| Males | Honey (23.5) *vs*. Water + honey (57.5) | 15.08 | 1 | 0.0001*** |
|  | Honey (23.5) *vs*. Water (10) | 13.02 | 1 | 0.0003*** |
|  | Honey (23.5) *vs*. Fasting (10) | 10.94 | 1 | 0.0009*** |
|  | Water (10) *vs*. Water + honey (57.5) | 19.89 | 1 | <0.0001**** |
|  | Water (10) *vs*. Fasting (10) | 0.5688 | 1 | 0.4507 NS |
|  | Fasting (10) *vs*. Water + honey (57.5) | 18.15 | 1 | <0.0001**** |
| Females | Honey (34) *vs*. Water + honey (68) | 7.577 | 1 | 0.0059** |
|  | Honey (34) *vs*. Water (9) | 30.35 | 1 | <0.0001**** |
|  | Honey (34) *vs*. Fasting (9) | 26.01 | 1 | <0.0001**** |
|  | Water (9) *vs*. Water + honey (68) | 33.73 | 1 | <0.0001**** |
|  | Water (9) *vs*. Fasting (9) | 1.724 | 1 | 0.1892 NS |
|  | Fasting (9) *vs*. Water + honey (68) | 28.92 | 1 | <0.0001**** |
| Females + hosts | Honey (30) *vs*. Water + honey (40) | 4.71 | 1 | 0.0300* |
|  | Honey (30) *vs*. Water (28) | 0.26 | 1 | 0.6101 NS |
|  | Honey (30) *vs*. Fasting (26) | 2.283 | 1 | 0.1308 NS |
|  | Water (28) *vs*. Water + honey (40) | 5.731 | 1 | 0.0167* |
|  | Water (28) *vs*. Fasting (26) | 1.345 | 1 | 0.2461 NS |
|  | Fasting (26) *vs*. Water + honey (40) | 7.742 | 1 | 0.0054** |
| Honey | Females (34) *vs*. Females + hosts (30) | 7.167 | 1 | 0.0074** |
| Water | Females (9) *vs*. Females + hosts (28) | 23.99 | 1 | <0.0001**** |
| Water + honey | Females (68) *vs*. Females + hosts (40) | 10.57 | 1 | 0.0107* |
| Fasting | Females (9) *vs*. Females + hosts (26) | 24.55 | 1 | <0.0001**** |
| Honey | Males (23.5) *vs*. Females (34) | 9.004 | 1 | 0.0027** |
| Water | Males (10) *vs*. Females (9) | 2.172 | 1 | 0.1406 NS |
| Water + honey | Males (57.5) *vs*. Females (68) | 1.350 | 1 | 0.2454 NS |
| Fasting | Males (10) *vs*. Females (9) | 4.368 | 1 | 0.0366* |
| Honey | Males (23.5) *vs*. Females + hosts (30) | 1.763 | 1 | 0.1843 NS |
| Water | Males (10) *vs*. Females + hosts (28) | 19.93 | 1 | <0.0001**** |
| Water + honey | Males (57.5) *vs*. Females + hosts (40) | 9.627 | 1 | 0.0019** |
| Fasting | Males (10) *vs*. Females + hosts (26) | 23.01 | 1 | <0.0001**** |
| **Assay #2 – Limited supply** |  |  |  |  |
| Females | Water + honey (6) *vs*. Water (4) | 107.2 | 1 | <0.0001**** |
|  | Water + honey (6) *vs*. Honey (5) | 22.61 | 1 | <0.0001**** |
|  | Water + honey (6) *vs*. Host (5) | 19.38 | 1 | <0.0001**** |
|  | Water + honey (6) *vs.* Fasting (3) | 92.14 | 1 | <0.0001**** |
|  | Water (4) *vs.* Honey (5) | 26.73 | 1 | <0.0001**** |
|  | Water (4) *vs*. Host (5) | 43.34 | 1 | <0.0001**** |
|  | Water (4) *vs.* Fasting (3) | 2.49 | 1 | 0.1148 NS |
|  | Honey (5) *vs.* Host (5) | 0.4941 | 1 | 0.4821 NS |
|  | Honey (5) *vs.* Fasting (3) | 32.32 | 1 | <0.0001**** |
|  | Host (5) *vs.* Fasting (3) | 44.78 | 1 | <0.0001**** |

^1^Values between parenthesis indicate median survival.

*P≤0.05, **P≤0.01, ***P≤0.001, ****P<0.0001, NS= non-significant difference.
