# Supplementary material for: Factors affecting the biology of Pachycrepoideus vindemmiae (Hymenoptera: Pteromalidae), a parasitoid of spotted-wing drosophila (Drosophila suzukii)
Source: PLoS One. 2019 Jul 23;14(7):e0218301. doi: 10.1371/journal.pone.0218301 (PMC6650059; doi:10.1371/journal.pone.0218301)
Supplement: S2 Table — N = 11 (water), 14 (honey), 10 (water + honey), and 12 (fasting). Each replicate was formed by one mated individual wasp. (DOCX) [file pone.0218301.s002.docx]

**S2 Table**

| **Biological parameter/normality test** | **Fasting** | **Water** | **Honey** | **Water + honey** |
| --- | --- | --- | --- | --- |
| **Fecundity – Entire adulthood** |  |  |  |  |
| D'Agostino & Pearson normality test |  |  |  |  |
| K2 | 0.3610 | 0.2618 | 1.990 | 7.023 |
| P value | 0.8349 | 0.8773 | 0.3697 | 0.0298 |
| Passed normality test (alpha=0.05)? | Yes | Yes | Yes | No |
| P value summary | ns | ns | ns | * |
|  |  |  |  |  |
| Shapiro-Wilk normality test |  |  |  |  |
| W | 0.9501 | 0.9736 | 0.8658 | 0.8687 |
| P value | 0.5996 | 0.9204 | 0.0459 | 0.1464 |
| Passed normality test (alpha=0.05)? | Yes | Yes | No | Yes |
| P value summary | ns | ns | * | ns |
|  |  |  |  |  |
| KS normality test |  |  |  |  |
| KS distance | 0.1672 | 0.1799 | 0.2505 | 0.2699 |
| P value | >0.1000 | >0.1000 | 0.0251 | 0.0890 |
| Passed normality test (alpha=0.05)? | Yes | Yes | No | Yes |
| P value summary | ns | ns | * | ns |
|  |  |  |  |  |
| **Emergence rate – Entire adulthood** |  |  |  |  |
| D'Agostino & Pearson normality test |  |  |  |  |
| K2 | 3.030 | 0.9713 | 0.03632 | 1.835 |
| P value | 0.2199 | 0.6153 | 0.9820 | 0.3995 |
| Passed normality test (alpha=0.05)? | Yes | Yes | Yes | Yes |
| P value summary | ns | ns | ns | ns |
|  |  |  |  |  |
| Shapiro-Wilk normality test |  |  |  |  |
| W | 0.9450 | 0.9511 | 0.9764 | 0.9503 |
| P value | 0.5251 | 0.6581 | 0.9487 | 0.6725 |
| Passed normality test (alpha=0.05)? | Yes | Yes | Yes | Yes |
| P value summary | ns | ns | ns | ns |
|  |  |  |  |  |
| KS normality test |  |  |  |  |
| KS distance | 0.1548 | 0.1790 | 0.1290 | 0.1567 |
| P value | >0.1000 | >0.1000 | >0.1000 | >0.1000 |
| Passed normality test (alpha=0.05)? | Yes | Yes | Yes | Yes |
| P value summary | ns | ns | ns | ns |

Cont…

**S2 Table (cont.)**

| **Biological parameter/normality test** | **Fasting** | **Water** | **Honey** | **Water + honey** |
| --- | --- | --- | --- | --- |
| **Sex ratio – Entire adulthood** |  |  |  |  |
| D'Agostino & Pearson normality test |  |  |  |  |
| K2 | 0.4967 | 1.263 | 0.7865 | 2.068 |
| P value | 0.7801 | 0.5319 | 0.6749 | 0.3556 |
| Passed normality test (alpha=0.05)? | Yes | Yes | Yes | Yes |
| P value summary | ns | ns | ns | ns |
|  |  |  |  |  |
| Shapiro-Wilk normality test |  |  |  |  |
| W | 0.9522 | 0.9067 | 0.9593 | 0.8478 |
| P value | 0.6323 | 0.2230 | 0.7113 | 0.0547 |
| Passed normality test (alpha=0.05)? | Yes | Yes | Yes | Yes |
| P value summary | ns | ns | ns | ns |
|  |  |  |  |  |
| KS normality test |  |  |  |  |
| KS distance | 0.1097 | 0.1934 | 0.1124 | 0.2115 |
| P value | >0.1000 | >0.1000 | >0.1000 | >0.1000 |
| Passed normality test (alpha=0.05)? | Yes | Yes | Yes | Yes |
| P value summary | ns | ns | ns | ns |
|  |  |  |  |  |
| **Miscellaneous attack – Entire adulthood** | | | | |
| D'Agostino & Pearson normality test |  |  |  |  |
| K2 | 5.120 | 0.9788 | 0.2714 | 1.124 |
| P value | 0.0773 | 0.6130 | 0.8731 | 0.5701 |
| Passed normality test (alpha=0.05)? | Yes | Yes | Yes | Yes |
| P value summary | ns | ns | ns | ns |
|  |  |  |  |  |
| Shapiro-Wilk normality test |  |  |  |  |
| W | 0.8214 | 0.9646 | 0.9760 | 0.9203 |
| P value | 0.0124 | 0.8278 | 0.9545 | 0.3591 |
| Passed normality test (alpha=0.05)? | No | Yes | Yes | Yes |
| P value summary | * | ns | ns | ns |
|  |  |  |  |  |
| KS normality test |  |  |  |  |
| KS distance | 0.2422 | 0.1840 | 0.1180 | 0.1722 |
| P value | 0.0357 | >0.1000 | >0.1000 | >0.1000 |
| Passed normality test (alpha=0.05)? | No | Yes | Yes | Yes |
| P value summary | * | ns | ns | ns |

Cont…

**S2 Table (cont.)**

| **Biological parameter/normality test** | **Fasting** | **Water** | **Honey** | **Water + honey** |
| --- | --- | --- | --- | --- |
| **Fecundity – Early adulthood (4-9 days old)** | | | | |
| D'Agostino & Pearson normality test | | | | |
| K2 | 3.026 | 1.181 | 0.1769 | 0.4867 |
| P value | 0.2202 | 0.5541 | 0.9154 | 0.7840 |
| Passed normality test (alpha=0.05)? | Yes | Yes | Yes | Yes |
| P value summary | ns | ns | ns | ns |
|  |  |  |  |  |
| Shapiro-Wilk normality test |  |  |  |  |
| W | 0.9218 | 0.9250 | 0.9642 | 0.9550 |
| P value | 0.2650 | 0.3625 | 0.7912 | 0.7274 |
| Passed normality test (alpha=0.05)? | Yes | Yes | Yes | Yes |
| P value summary | ns | ns | ns | ns |
|  |  |  |  |  |
| KS normality test |  |  |  |  |
| KS distance | 0.1925 | 0.1497 | 0.1151 | 0.1513 |
| P value | >0.1000 | >0.1000 | >0.1000 | >0.1000 |
| Passed normality test (alpha=0.05)? | Yes | Yes | Yes | Yes |
| P value summary | ns | ns | ns | ns |
|  |  |  |  |  |
| **Emergence rate – Early adulthood (4-9 days old)** | | | | |
| D'Agostino & Pearson normality test | | | | |
| K2 | 2.126 | 2.431 | 1.798 | 1.470 |
| P value | 0.3454 | 0.2965 | 0.4069 | 0.4795 |
| Passed normality test (alpha=0.05)? | Yes | Yes | Yes | Yes |
| P value summary | ns | ns | ns | ns |
|  |  |  |  |  |
| Shapiro-Wilk normality test |  |  |  |  |
| W | 0.8516 | 0.9192 | 0.9148 | 0.8726 |
| P value | 0.0299 | 0.3123 | 0.1847 | 0.1073 |
| Passed normality test (alpha=0.05)? | No | Yes | Yes | Yes |
| P value summary | * | ns | ns | ns |
|  |  |  |  |  |
| KS normality test |  |  |  |  |
| KS distance | 0.2848 | 0.1781 | 0.1907 | 0.2458 |
| P value | 0.0050 | >0.1000 | >0.1000 | 0.0882 |
| Passed normality test (alpha=0.05)? | No | Yes | Yes | Yes |
| P value summary | ** | ns | ns | ns |

Cont…

**S2 Table (cont.)**

| **Biological parameter/normality test** | **Fasting** | **Water** | **Honey** | **Water + honey** |
| --- | --- | --- | --- | --- |
| **Sex ratio – Early adulthood (4-9 days old)** | | | | |
| D'Agostino & Pearson normality test | | | | |
| K2 | 1.017 | 3.478 | 12.41 | 6.009 |
| P value | 0.6015 | 0.1757 | 0.0020 | 0.0496 |
| Passed normality test (alpha=0.05)? | Yes | Yes | No | No |
| P value summary | ns | ns | ** | * |
|  |  |  |  |  |
| Shapiro-Wilk normality test |  |  |  |  |
| W | 0.8893 | 0.8793 | 0.8348 | 0.8744 |
| P value | 0.0954 | 0.1019 | 0.0139 | 0.1371 |
| Passed normality test (alpha=0.05)? | Yes | Yes | No | Yes |
| P value summary | ns | ns | * | ns |
|  |  |  |  |  |
| KS normality test |  |  |  |  |
| KS distance | 0.1684 | 0.2264 | 0.2012 | 0.1759 |
| P value | >0.1000 | >0.1000 | >0.1000 | >0.1000 |
| Passed normality test (alpha=0.05)? | Yes | Yes | Yes | Yes |
| P value summary | ns | ns | ns | ns |
|  |  |  |  |  |
| **Miscellaneous attack – Early adulthood (4-9 days old)** | | | | |
| D'Agostino & Pearson normality test | | | | |
| K2 | 0.7247 | 1.493 | 2.506 | 0.3220 |
| P value | 0.6960 | 0.4739 | 0.2856 | 0.8513 |
| Passed normality test (alpha=0.05)? | Yes | Yes | Yes | Yes |
| P value summary | ns | ns | ns | ns |
|  |  |  |  |  |
| Shapiro-Wilk normality test |  |  |  |  |
| W | 0.9441 | 0.9128 | 0.9063 | 0.9783 |
| P value | 0.5525 | 0.2631 | 0.1634 | 0.9551 |
| Passed normality test (alpha=0.05)? | Yes | Yes | Yes | Yes |
| P value summary | ns | ns | ns | ns |
|  |  |  |  |  |
| KS normality test |  |  |  |  |
| KS distance | 0.1443 | 0.1740 | 0.1928 | 0.1480 |
| P value | >0.1000 | >0.1000 | >0.1000 | >0.1000 |
| Passed normality test (alpha=0.05)? | Yes | Yes | Yes | Yes |
| P value summary | ns | ns | ns | ns |
