# Supplementary material for: Factors affecting the biology of Pachycrepoideus vindemmiae (Hymenoptera: Pteromalidae), a parasitoid of spotted-wing drosophila (Drosophila suzukii)
Source: PLoS One. 2019 Jul 23;14(7):e0218301. doi: 10.1371/journal.pone.0218301 (PMC6650059; doi:10.1371/journal.pone.0218301)
Supplement: S3 Table — N = 11 (water), 14 (honey), 10 (water + honey), and 12 (fasting). Each replicate was formed by one mated individual wasp. (DOCX) [file pone.0218301.s003.docx]

**S3 Table**

| **Biological parameter/homoscedasticity test** | |
| --- | --- |
| **Fecundity – Entire adulthood** |  |
| Brown-Forsythe test |  |
| F (DFn, DFd) | 1.003 (3, 41) |
| P value | 0.4012 |
| P value summary | ns |
| Are SDs significantly different (P < 0.05)? | No |
|  |  |
| Bartlett's test |  |
| Bartlett's statistic (corrected) | 5.147 |
| P value | 0.1613 |
| P value summary | ns |
| Are SDs significantly different (P < 0.05)? | No |
|  |  |
| **Emergence rate – Entire adulthood** |  |
| Brown-Forsythe test |  |
| F (DFn, DFd) | 1.019 (3, 44) |
| P value | 0.3935 |
| P value summary | ns |
| Are SDs significantly different (P < 0.05)? | No |
|  |  |
| Bartlett's test |  |
| Bartlett's statistic (corrected) | 5.218 |
| P value | 0.1565 |
| P value summary | ns |
| Are SDs significantly different (P < 0.05)? | No |
|  |  |
| **Sex ratio – Entire adulthood** |  |
| Brown-Forsythe test |  |
| F (DFn, DFd) | 1.618 (3, 44) |
| P value | 0.1989 |
| P value summary | ns |
| Are SDs significantly different (P < 0.05)? | No |
|  |  |
| Bartlett's test |  |
| Bartlett's statistic (corrected) | 5.674 |
| P value | 0.1286 |
| P value summary | ns |
| Are SDs significantly different (P < 0.05)? | No |

Cont…

**S3 Table (cont.)**

| **Biological parameter/homoscedasticity test** | |
| --- | --- |
| **Miscellaneous attack – Entire adulthood** | |
| Brown-Forsythe test |  |
| F (DFn, DFd) | 0.6700 (3, 43) |
| P value | 0.5751 |
| P value summary | ns |
| Are SDs significantly different (P < 0.05)? | No |
|  |  |
| Bartlett's test |  |
| Bartlett's statistic (corrected) | 5.378 |
| P value | 0.1461 |
| P value summary | ns |
| Are SDs significantly different (P < 0.05)? | No |
|  |  |
| **Fecundity – Early adulthood (4-9 days old)** | |
| Brown-Forsythe test |  |
| F (DFn, DFd) | 1.270 (3, 44) |
| P value | 0.2965 |
| P value summary | ns |
| Are SDs significantly different (P < 0.05)? | No |
|  |  |
| Bartlett's test |  |
| Bartlett's statistic (corrected) | 4.315 |
| P value | 0.2294 |
| P value summary | ns |
| Are SDs significantly different (P < 0.05)? | No |
|  |  |
| **Emergence rate – Early adulthood (4-9 days old)** | |
| Brown-Forsythe test |  |
| F (DFn, DFd) | 1.034 (3, 44) |
| P value | 0.3867 |
| P value summary | ns |
| Are SDs significantly different (P < 0.05)? | No |
|  |  |
| Bartlett's test |  |
| Bartlett's statistic (corrected) | 2.970 |
| P value | 0.3963 |
| P value summary | ns |
| Are SDs significantly different (P < 0.05)? | No |

Cont…

**S3 Table (cont.)**

| **Biological parameter/homoscedasticity test** | |
| --- | --- |
| **Sex ratio – Early adulthood (4-9 days old)** | |
| Brown-Forsythe test |  |
| F (DFn, DFd) | 1.385 (3, 43) |
| P value | 0.2603 |
| P value summary | ns |
| Are SDs significantly different (P < 0.05)? | No |
|  |  |
| Bartlett's test |  |
| Bartlett's statistic (corrected) | 10.17 |
| P value | 0.0172 |
| P value summary | * |
| Are SDs significantly different (P < 0.05)? | Yes |
| Brown-Forsythe test |  |
|  |  |
| **Miscellaneous attack – Early adulthood (4-9 days old)** | |
| F (DFn, DFd) | 1.148 (3, 41) |
| P value | 0.3411 |
| P value summary | ns |
| Are SDs significantly different (P < 0.05)? | No |
|  |  |
| Bartlett's test |  |
| Bartlett's statistic (corrected) | 3.525 |
| P value | 0.3176 |
| P value summary | ns |
| Are SDs significantly different (P < 0.05)? | No |
