# Supplementary material for: Factors affecting the biology of Pachycrepoideus vindemmiae (Hymenoptera: Pteromalidae), a parasitoid of spotted-wing drosophila (Drosophila suzukii)
Source: PLoS One. 2019 Jul 23;14(7):e0218301. doi: 10.1371/journal.pone.0218301 (PMC6650059; doi:10.1371/journal.pone.0218301)
Supplement: Attachment — Submitted filename: Response to reviewers.docx [file pone.0218301.s006.docx]

**Manuscript title: “Factors affecting the biology of *Pachycrepoideus vindemmiae* (Hymenoptera: Pteromalidae) on spotted-wing drosophila (*Drosophila suzukii*)”**

By Bezerra Da Silva et al.

**REBUTTAL LETTER**

**Reviewer #1**

**Reviewer:** “In Table 1, for instance, it is written: “Comparison of survival curves by long-rank (Mantel-Cox) test to evaluate the impact of constant and limited supply of water, honey, water + honey, and fasting (no honey, no water) on adult males and females of Pachycrepoideus vindemmiae (Hymenoptera: Pteromalidae), in the presence and absence of hosts…”, but when we look at the table, the results are separated by wasp’s sex and host presence, with no mention to any supply. Does that mean that the authors evaluated the effects of all supplies (water, honey, water + honey, and fasting) combined on males and females? By reading the corresponding result section, I don’t think so, since it is written: “…we found that the availability of water + honey resulted in significantly higher survival curves than honey alone, water alone, and fasting (no honey, no water), independent of sex and host availability (Males: P<0.0001, N=8-9; Females: P<0.0001, 129 N=10-16; Females + host: P=0.0049, N=9-15;) (Fig 2ab, Table 1). Here they discriminated the treatments, although Table 1 had not separated effects. It seems that the Female + host treatment (P=0.0049) considered the effects of water + honey in comparison to the other treatments (at least it is the same P value in the table). Furthermore, if they are comparing the water + honey treatment with the other three, it should not there be three P values for each sex treatment? (Female: water + honey x honey; water + honey x water; water + honey x fasting; Male: water + honey x honey…). The treatments got me confused, too. In the methods section, the authors established (for assay 1) eight treatments: (1) water, (2) honey, (3) water + honey, (4) no water, no honey (fasting), (5) water + hosts, (6) honey + hosts, (7) water + honey + hosts, and (8) no water, no honey + hosts. But in Table 1 the “hosts” treatments are not considered as supply, but in a treatment called Female + hosts. Although I believe that the authors did that since only females had the “host” treatment, it seems the treatments are changing throughout the text.”

**Authors:** We recognize that the way we presented the data is confusing and we apologize for it. You are right. The results for Assay #1 in Table 1 actually show the effect of diet (water, honey, water + honey, no water + no honey) on the survival curves of males, females, and females + hosts. That is why it shows 3 P-values: the impact of diet on the survival curves was evaluated separately for each one of those three parasitoid groups. In the Assay #2, we evaluated the effect of a set of diets that was different from Assay #1 (i.e., water, honey, water + honey, no water + no honey, and ***host***). So, in order to avoid confusion, we are eliminating the results for Assay #2 from Table 1 and inserting them in the main text. Additionally, we have reworded the title of the table to make it clearer.

**Reviewer:** “I also cannot understand the N used in the experiments. Most of the times the N shown are intervals. In Table 1 we have N=8-9; N=10-16 and N=9-15. What does an N of 9-15 mean? The reader must know exactly the N of each experiment.”

**Authors:** “N” is the number of replicates (=individual mated adult wasps) in each treatment. For example, for males, we had 10, 8, 9, and 8 replicates respectively for the water, honey, water + honey, and no water + no honey treatments. Hence, an N of 8-10 means that the number of replicates of the treatments in a given group (e.g., males) ranged from 8 to 10. We agree with you when you say that the “reader must know exactly the N of each experiment”. So we are providing that information in the titles of all tables and figures.

**Reviewer:** “In Table 3, the legend reports an N = 8-16. However, the authors reported a DFd of 40 to 44, which means that the N was of at least 42 (DFd = N – a, where a = number of factors, which is 2, I believe). How are those values so different? Maybe there was some kind of pseudoreplication?”

**Authors:** The “DFd of 40 to 44” happens because the analysis take into consideration all replicates of all 4 treatments (water, honey, water + honey, and no water + no honey). We have included the actual number of replicates of each treatment in the title of Table 3.

**Reviewer:** “In Table 2, the host diet is “not applicable” to any treatments (male, female and female + hosts). I do not understand why, since the authors used hosts in half of the treatments described in assay 1, and there is even a treatment here that considered hosts. Is that because the authors did not offer only hosts in any treatment (assay 1)? If that is so, why is this treatment observed in assay 2?

**Authors:** We agree that Table 2 is confusing. We used “n/a” in all adult groups (male, female, and female + hosts) of Assay #1 for different reasons:

1. In the “Male” column, “n/a” was used because males were never offered hosts.
2. In the “Female” column, “n/a” was used because the purpose of this treatment was to investigate the impact of diet (water, honey, water + honey, and no water + no honey) on host-deprived females.
3. In the “Female + hosts” column, “n/a” was used because hosts were offered along with all treatments (diets) in this column. So, it wouldn’t make sense to offer hosts to insects that had already been offered hosts.

In order to avoid confusion, we have transferred the results for assay #2 from Table 2 to the main text in the Results section. Since then, Table 2 has been dedicated exclusively to assay #1.

**Reviewer:** “Also, why did the authors choose to conduct two assays? I saw no justification for that.”

**Authors:** We needed two bioassays because we wanted to investigate the impact of constant (assay #1) and limited (assay #2) supplies of resources on the parasitoid.

**Reviewer:** “The abstract needs some improvements. The use of ‘host” as supply (together with water and honey) is confusing since I did not imagine that adults of P. vindemmiae feed on its hemolymph; I was thinking about the eggs inserted in the hosts. It should be clear that the adults feed on the pupae. This confusion happens during the text because sometimes it is not clear whether the authors are writing about the adult or juvenile wasps. I had to read the introduction and methods to understand the abstract.

**Authors:** After you comment, we have clarified that the parasitoid host-feeds on the hemolymph of SWD pupae in the abstract.

**Reviewer:** “In addition, do the adults kill the pupae when they start to feed on it? Maybe this can be clarified in the introduction.”

**Authors:** Yes, SWD pupae that whose hemolymph has been drunk by *P. vindemmiae* can die. We have clarified that in the Introduction.

**Reviewer:** “To conclude, I consider this manuscript to be promising, but the presentation of methods and results made me struggle to understand it. Although I see no major problems in the introduction and discussion, the manuscript still needs a lot of work and the ideas need to be more clear.”

**Authors:** We have performed many improvements and clarifications in the methods and results in order to make the manuscript more clear. Thank you very much for your suggestions and helpful criticism.

**Reviewer:** “By reading the title, it is not possible to understand that the system involves a parasitoid species unless the reader knows the involved species. I had to read the abstract to understand it. I suggest the authors let this information explicit.”

**Authors:** The suggested changes have been implemented.

**Reviewer:** “Line 33: Does “host-killing” mean the offspring kill? It would be good to define this term. I was confusing it with “host-feeding”.”

**Authors:** It means the parasitoid kills, independent of its developmental stage. We have clarified that in the text.

**Reviewer:** “Line 53: It needs a reference.”

**Authors:** References have been provided (9-11).

**Reviewer:** “Lines 100-103: It seems a result part. If not, it needs a reference.”

**Authors:** The reference 42 is provided for lines 100-105.

**Reviewer:** “Lines 114-116: This sentence needs to be justified.”

**Authors:** It has been justified in the lines 65-68.

**Reviewer:** “Line 159: Is the ± SD, SE?”

**Authors:** It is max and min temperature and R.H. measured during our experiments.

**Reviewer:** “Line 200: No need of the word parametric here. ANOVA is ways a parametric test.

**Authors:** That is not very accurate. For example: <https://www.youtube.com/watch?v=butN8JLWhyE> and also: <https://en.wikipedia.org/wiki/Kruskal%E2%80%93Wallis_one-way_analysis_of_variance>

**Reviewer:** “Lines 204-205: The table needs to be formatted.”

**Authors:** Unfortunately, we did not understand specifically you want us to format. Additional changes was made as suggested by other reviewers.

**Reviewer:** “Lines 225-226: Should they be 0.4589 and 0.1835?”

**Authors:** No. They should be 0.2613 and 0.4589, as shown in Table 3. We have corrected them.

**Reviewer:** “Lines 232-233: Is that the honey and water treatments alone or it includes water + honey treatment?”

**Authors:** Those are actually the results of the two-way anova, i.e., the results for the factor honey (absence or presence) and for the factor water (absence or presence). We have clarified that in the text.

**Reviewer:** “Line 253: If that is the standard error of the mean, it should be SE instead.”

**Authors:** Unfortunately, we have to disagree: <https://en.wikipedia.org/wiki/Standard_error>

**Reviewer:** “Line 257: Is that ± SE?”

**Authors:** It is just the temperature and R.H. variation (max. and min.), as explained before.

**Reviewer:** “Line 269: 6-11 days cannot be on average.”

**Authors:** We have eliminated “on average”.

**Reviewer:** “Line 293: Why does it have intervals here (0.5-1; 17-19)?”

**Authors:** Because we had 5 treatments here (water, honey, water + honey, fasting, and host). The intervals represent the min. and max. values found amongst those five treatments.

**Reviewer:** “Lines 346-347: The first half of the sentence has an odd construction.”

**Authors:** We have reworded the sentence.

**Reviewer:** “Lines 354-357: I do not understand what do you mean here. Do you mean that there is no trade-off since the wasp does not kill the host?”

**Authors:** No. We mean that there is no trade-off since host-feeding does not affect parasitoid offspring. We have reworded the sentence for clarification.

**Reviewer:** “Line 356: I saw both “trade-off” and “tradeoff” (line 375) throughout the manuscript. Please standardize.”

**Authors:** Done.

**Reviewer:** “Lines 383-386: it would be more informative if you explain right away that the water deprivation (not water availability) increased the host-killing.”

**Authors:** Done.

**Reviewer:** “Lines 388-391: Although the diet has not affected the emergence rates, it affected the number of viable eggs (Lines 327-330). Maybe these two parts can be gathered.”

**Authors:** Thanks for the suggestion. But because in lines 327-330 we meant the ability of females to lay eggs at all, and in lines 388-391 we meant viable eggs (i.e., eggs that were laid but did not give rise to larvae), we decided not to gather the two sentences. We have clarified that in the text.

**Reviewer:** “Lines 152, 155, 250, 539, 756: Please fix the term to “log-rank”.”

**Authors:** Done.

**Reviewer #2**

**Reviewer:** “I missed a hypothesis indicating what they would expect given that they performed treatments with individuals supplying and omitting different resources.”

**Authors:** Done. We have more clearly stated the hypotheses in the Introduction.

**Reviewer:** “Line 175-177: "Fecundity was significantly increased by water deprivation (honey and fasting)..., while the effect of honey was insignificant". I think that you missed to discuss these results (these are even mentioned in line 31-33 of the abstract), so I believe that it is necessary to discuss why water is increased fecundity in young females and honey was not shown significant differences, what could be the reason for that.”

**Authors:** You are right. However, we have dedicated a different study to investigate and discuss that matter:

Bezerra Da Silva CS, Price BE, Walton VM. Water-Deprived Parasitic Wasps (*Pachycrepoideus vindemmiae*) Kill More Pupae of a Pest (*Drosophila suzukii*) as a Water-Intake Strategy. Scientific Reports. 2019;9: 3592. doi:10.1038/s41598-019-40256-8

In the current manuscript, we focused our discussion in the differences found between early and entire female adulthood. We therefore included the above-mentioned reference in the lines 175-177 in order to warn the reader that there is one entire study focusing on the matter of “water-deprivation increasing fecundity”.

**Reviewer:** “Line 253: Table 2 is after table 3, which is in line 200, you need to place the tables in an ordered numbering.”

**Authors:** Done.

**Reviewer:** “Line 467: "Emerged adults were released into new Bugdorm cages to restart the cycle"... Were individuals placed in new cages separately by sex? Or together? Please specify this.”

**Authors:** Done. Males and females were mixed together in new cages.

**Reviewer:** “Line 486: “We tested 8-14 wasp/treatment/sex”. Could you describe it better. Were 8 to 14 wasps tested per treatment and per sex? Was that what you meant? That's what I understood.”

**Authors:** Yes, that is exactly what we meant. We have clarified that in the text.

**Reviewer:** “Line 490-492: "To control for potential differences in the relative humidity among treatments that were water deprived (1, 3, 5, and 7) and water provided (2, 4, 6, and 8)". The deprived water treatments were not 2,4,6,8 and those supplied with water were 1,3,5,7? that was confusing. On line 474 you mention the kinds of treatment and there you state that 1, 3, 5 and 7 were supplied with water and the other ones were deprived of water.”

**Authors:** Nice catch! You are totally right! We are sorry for the confusion. We have performed corrections in the text.

**Reviewer:** “Line 515 - 520: In my view, I recommend that for a better understanding this paragraph should be at the beginning, in line 472, because if not, it sounds like you're coming back on a subject that you've already explained and with no order in the text.”

**Authors:** Here we have to disagree with you. First we have to explain which biological parameters we are measuring and then explain that we are measuring those parameters for early and entire adulthood, and then explaining why we are doing so. If we do that in the first paragraph, before we even explain the basics, then we think it would seems like there is no order in the text.

**Reviewer:** “Supporting information - S1_table: I consider that means, medians and variances of the data are not included, as well as the normality and homocedasticity tests of the analyzed data.”

**Authors:** Following your suggestion we have added the medians to S1 Table. S2 Table and S3 Table have also been created to show the results for normality and homoscedasticity tests, respectively.
